# Metal-phenolic networks: facile assembled complexes for cancer theranostics

**DOI:** 10.7150/thno.58711

**Published:** 2021-04-19

**Authors:** Wensheng Xie, Zhenhu Guo, Lingyun Zhao, Yen Wei

**Affiliations:** 1 The Key Laboratory of Bioorganic Phosphorus Chemistry & Chemical Biology (Ministry of Education), Department of Chemistry, Tsinghua University, Beijing 100084, P. R. China.; 2 State Key Laboratory of New Ceramics and Fine Processing, School of Materials Science and Engineering, Tsinghua University, Beijing 100084, China.

**Keywords:** metal-phenolic network, self-assembly, biocompatibility, cancer theranostics, nanoagent

## Abstract

In recent years, metal-phenolic networks (MPNs) have attracted increasing attention for the engineering of multi-functional platforms because of their easy fabrication processes, excellent physicochemical properties, outstanding biocompatibility, and promising theranostic applications. In this review, we summarize recent progress in the design, synthesis, shape-control, biocompatibility evaluation, and potential theranostic applications of MPNs, especially for cancer theranostics. First, we provide an overview of various MPN systems, relevant self-assembly procedures, and shape-controllable preparation. The *in vitro* and *in vivo* biocompatibility evaluation of MPNs is also discussed, including co-incubation viability, adhesion, bio-distribution, and inflammation. Finally, we highlight the significant achievements of various MPNs for cancer theranostics, such as tumor imaging, drug delivery, photothermal therapy, radiotherapy, and chemo- and photo-dynamic therapy. This review provides a comprehensive background on the design and controllable synthesis, *in vitro* and *in vivo* biocompatibility evaluation, applications of MPNs as cancer theranostic agents, and presents an overview of the most up-to-date achievements in this field.

## Introduction

**Figure 1:**
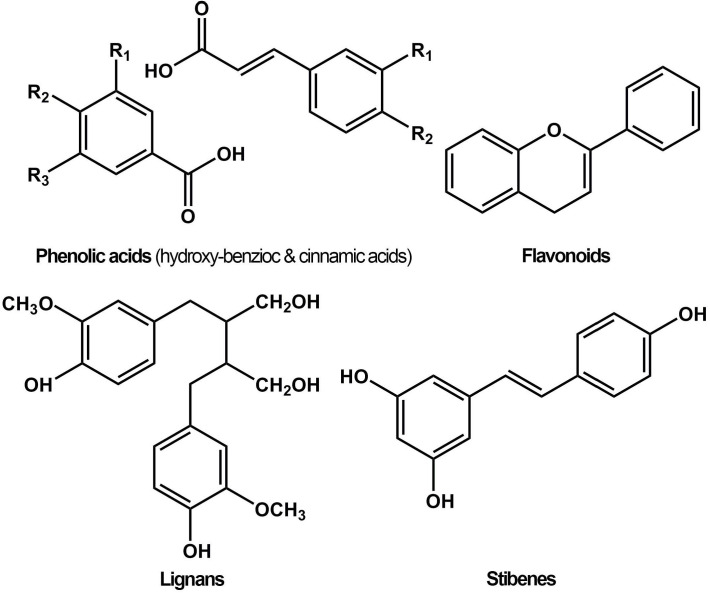
Chemical structures of different groups of polyphenols.

Phenols, also called phenolics, are a class of organic compounds consisting of one or more hydroxyl (-*OH*) groups directly attached to an aromatic hydrocarbon group. Phenols and polyphenols normally exist in plant foods such as vegetables, fruits, spices, herbs, tea, and wine, and are associated with various biological functions, including chemical defense, structural support, metal sequestration, pigmentation, and prevention of radiation damage 1. Based on the chemical structure, natural polyphenols are categorized into four main groups, namely flavonoids, lignans, phenolic acids, and stilbenes (**Figure 1**) 2. Among these, phenolic acids and flavonoids, accounting for about 30% and 60% respectively, are the most common groups. Flavonoids are categorized into anthocyanins, flavanols, flavanones, flavones, flavonols, and isoflavonoids. Common phenolic acids include hydroxybenzoic acid (ellagic acid, gallic acid) and hydroxycinnamic acid (ferulic acid, chlorogenic acid).

Phenols (and polyphenols) possess various cardioprotective, anti-inflammatory, anti-aging, antioxidant, anticancer, and antimicrobial activities, have kidney protective, lung protective, and neuroprotective effects, and are also effective against UV irradiation and prevention of osteoporosis 3,4. Polyphenols can act as antioxidants, neutralizing harmful free radicals to avoid cellular damage and decrease the risk of cancer, diabetes and heart diseases 5. A plethora of studies have demonstrated the anticancer effects of polyphenols due to their potent antioxidant and anti-inflammatory activities and the ability to modulate signaling pathways and molecular targets 3.

Polyphenols are excellent raw materials to prepare multi-functional inorganic-organic hybrids for biomedical applications and are considered potential candidates for applications in many fields, such as energy, transportation, optics, microelectronics, environment, housing, health and diagnostics 6. Specifically, the self-assembly coordination of organic ligands with functional metal species makes a critical difference in biomedical functions. The galloyl and catechol groups of phenols act as multivalent chelating sites with pH responsiveness for coordinating with various metals like Fe^III^ 7. Therefore, metal-phenolic networks (MPNs) have attracted attention in the fields of nanomaterials, biointerfaces, particle engineering, tunable targeted delivery systems, and anti-cancer treatment.

MPNs are super-molecular network structures consisting of metal ions coordinated with phenolic ligands. Compared to metal organic frameworks (MOFs) or other metal polymer complexes, MPNs are environment-friendly for the green phenolic ligands 8. One of the significant advantages of MPNs is that they could act as coating materials for surface modifications by attaching to diverse substrates regardless of the size, construction and composition 9. For example, Liu et al. reported a polymeric/inorganic nanoparticle/nanovesicle-supported Fe^III^-tannic acid (TA) platform for tumor-specific photoactivated utilizations, including *T*_1_-weighted magnetic resonance imaging (*T*_1_-MRI imaging), photoacoustic imaging, photo-thermal imaging together with NIR photo-thermal therapy with complete tumor elimination 10. Herein, we describe simple preparation processes via self-assembly coordination with advanced performances and present a full panoramic sketch of MPNs on cancer theranostics that may inspire further research. First section will introduce various systems and procedures for MPN synthesis, various shape-controllable preparation methods, and biocompatibility concerns for the *in vitro/in vivo* treatment. The second section of this review will discuss various cancer theranostic applications of MPNs, including tumor imaging, drug delivery systems for chemotherapy, photothermal therapy, radiotherapy, and chemo-/photodynamic therapy.

## Metal-Phenolic Networks

### Various MPN systems

**Figure 2:**
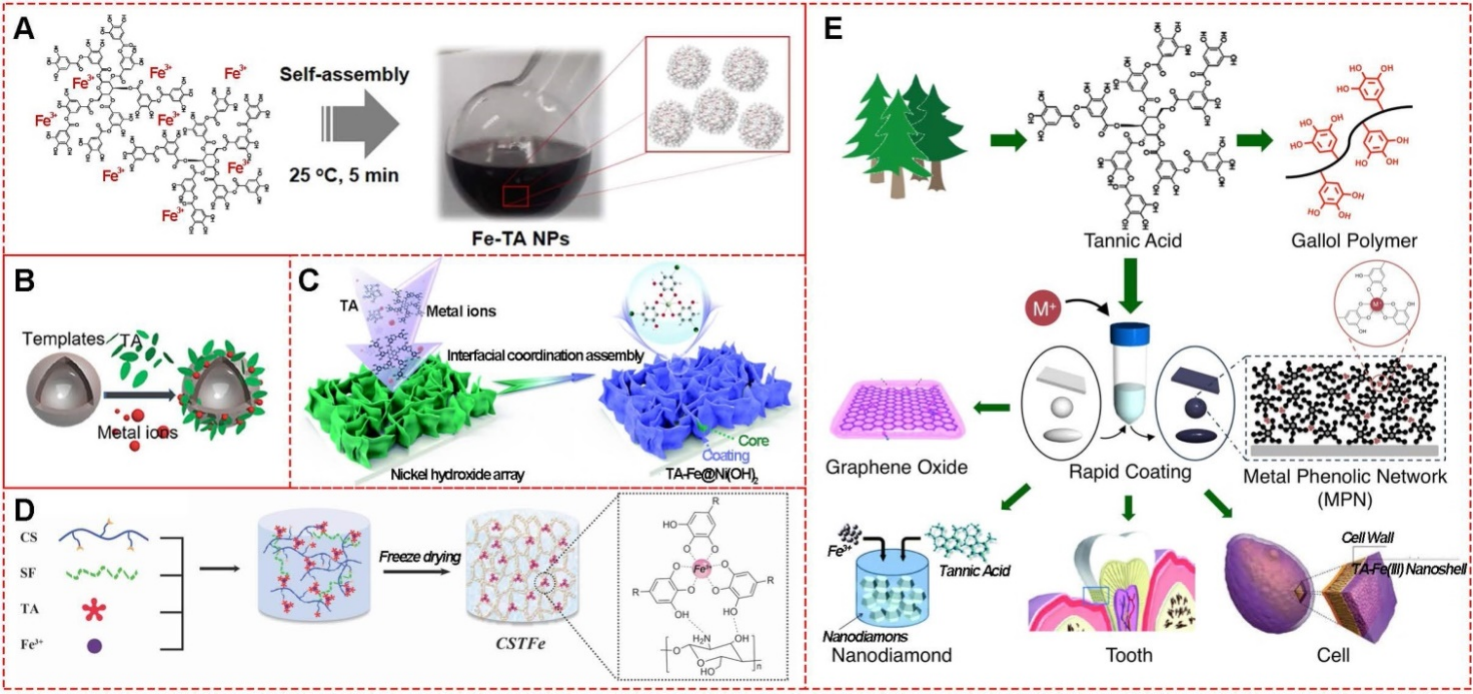
Various TA-based MPNs. (A) Fe^III^-TA nanoparticles. (B) Fe^III^-TA capsules. (C) Fe^III^-TA coating film. (D) Fe^III^-TA cryogel. (E) Various applications of TA-based MPNs. (A) Adapted with permission from 11, copyright 2018 Springer Nature Limited. (B) Adapted with permission from 12, copyright 2018 American Chemical Society. (C) Adapted with permission from 13, Copyright 2020 Royal Society of Chemistry. (D) Adapted with permission from 14, copyright 2019 Wiley Online Library. (E) Adapted with permission from 15, copyright 2016 Elsevier B.V.

MPNs consist of two parts: the central metal ions and phenolic ligands. A single organic ligand can coordinate with a variety of metals, or changing organic ligands for a specific metal ion are both significant strategies to design facile functionalized MPNs. The commonly used organic components are simple phenolic ligands, namely the monomeric building blocks of natural polyphenols 10. Among them, tannic acid (TA), an approved food additive by US Food and Drug Administration, plays a prominent role in the natural polyphenol family. The chelating sites provided by adjacent hydroxyl groups in TA can react with various metal ions. The abundant gallol groups on TA help coordination-driven cross-linking, leading to the production of three-dimensionally stabilized MPNs. Therefore, diverse MPNs based on TA for multi-functional applications have been widely explored in recent years (**Figure 2**). By simply mixing TA with Fe^III^ in phosphate-buffered saline for a few minutes at ambient temperature, large-scale Fe^III^-TA nanoparticles could be obtained with excellent physicochemical properties (**Figure 2A**) 11. Also, Fe^III^-TA capsule and film can be easily obtained by introducing different templates or substrates (**Figure 2B and 2C**) 12,13. One-step mixing with the natural silk fibroin, chitosan, TA, and Fe^III^ of a 3-dimensional multi-functional wound dressing cryogel (**Figure 2D**) has been described for efficient wound healing 14. Due to their unique physicochemical properties, TA-based MPNs were widely used as a versatile platform to engineer nanomaterials and bio-interfaces (**Figure 2E**) 15. For example, simple mixing of the doxorubicin-loaded dendrimer with tannic acid and Fe^III^ formed MPNs, and Den-DOX-tannic acid-Fe^III^ (DDTF) nanoparticles were obtained to overcome the multidrug resistance of tumor through the apoptosis/ferroptosis hybrid pathway 16. Furthermore, TA-based MPNs have been widely studied as drug delivery systems and anti-bacterial agents and in bone tissue regeneration applications, photodynamic therapy, chemo-dynamic therapy, wound repair for skin regeneration, and imaging tools for metal ions.

**Figure 3:**
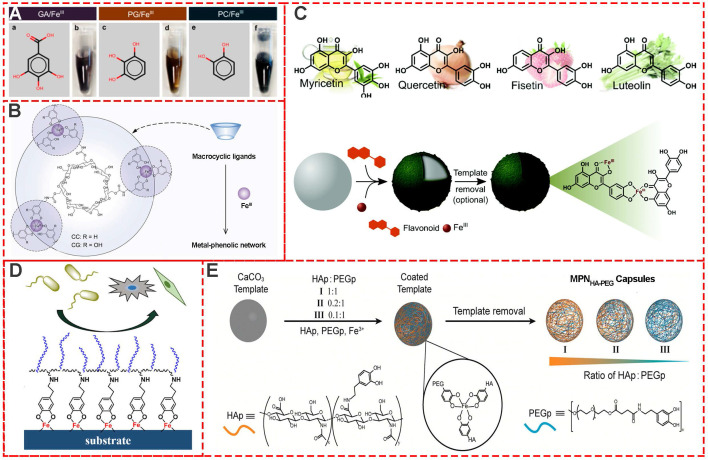
Various MPN systems based on different phenolic ligands. (A) Gallic acid (GA), pyrogallol (PG), and pyrocatechol (PC). (B) Cyclodextrin catechol (CC), cyclodextrin galloyl (CG). (C) Various flavonoids including myricetin, quercetin, fisetin, and luteolin. (D) Copolymerization of dopamine methacrylamide and poly (ethylene glycol) methyl methacrylate. (E) Phenolic-functionalized hyaluronic acid and phenolic-functionalized poly-ethylene glycol. (A) Adapted with permission from 17, copyright 2015 American Chemical Society. (B) Adapted with permission from 18, copyright 2019 Wiley Online Library. (C) Adapted with permission from 19, copyright 2017 Royal Society of Chemistry. (D) Adapted with permission from 20, copyright 2019 American Chemical Society. (E) Adapted with permission from 21, copyright 2016 American Chemical Society.

Besides TA-based MPNs, many other natural plant phenols, including pyrogallol (PG), gallic acid (GA), pyrocatechol (PC) (**Figure 3A**) 17, cyclodextrin catechol (CC), cyclodextrin galloyl (CG) (**Figure 3B**) 18, myricetin, quercetin, luteolin and fisetin (**Figure 3C**) 19 have also been successfully used as building blocks for MPNs. Moreover, to design and prepare multi-functional MPNs, modification of the phenolic ligands, such as polyphenol-functionalized polymers, is always an effective strategy. Modified phenolic ligands such as dopamine-functionalized methacrylamide, poly (ethylene glycol) methyl methacrylate (**Figure 3D**) 20, phenolic-functionalized hyaluronic acid (HAp), poly (ethylene glycol) (PEGp) (**Figure 3E**) 21, and catechol-functionalized chitosan (Chitosan-catechol) have been employed. Examples of other excellent research studies include self-healing hydrogel based on the self-assembly coordination of a 4-arm-poly (ethylene glycol) (4-arm-PEG)-catechol and Fe^III^ 22, PEG-MPN capsules by conjugating catechol groups onto each terminus of an eight-arm-PEG 23, and p(PEGMA-*co*-DMA)/Fe MPNs for antimicrobial and antifouling applications 24.

Besides the multi-functional modification of phenolic ligands, specific incorporation of metal ions into phenolic ligands based on the self-assembly metal-coordination interactions to impart functionalities has also been systematically examined. Guo et al. designed and prepared 18 MPNs through TA coordination with 18 different metal ions: manganese (Mn), iron (Fe), aluminum (Al), cobalt (Co), vanadium (V), copper (Cu), zinc (Zn), nickel (Ni), chromium (Cr), zirconium (Zr), molybdenum (Mo), rhodium (Rh), cerium (Ce), europium (Eu), cadmium (Cd), gadolinium (Gd), ruthenium (Ru), and terbium (Tb) 25. First, a substrate template was used to form MPN films by directly mixing metal solutions with TA. Subsequently, highly monodisperse capsules were obtained after the removal of the template. Further studies showed that the stability and thickness of the MPN films are influenced by the kind of metal and its specific feed concentration. Final results exhibited that Cu^II^-TA capsules were the thinnest and the Zr^IV^-TA capsules the thickest. These various MPN systems are summarized in **Table 1**.

## Self-assembly procedures for MPNs

**Figure 5:**
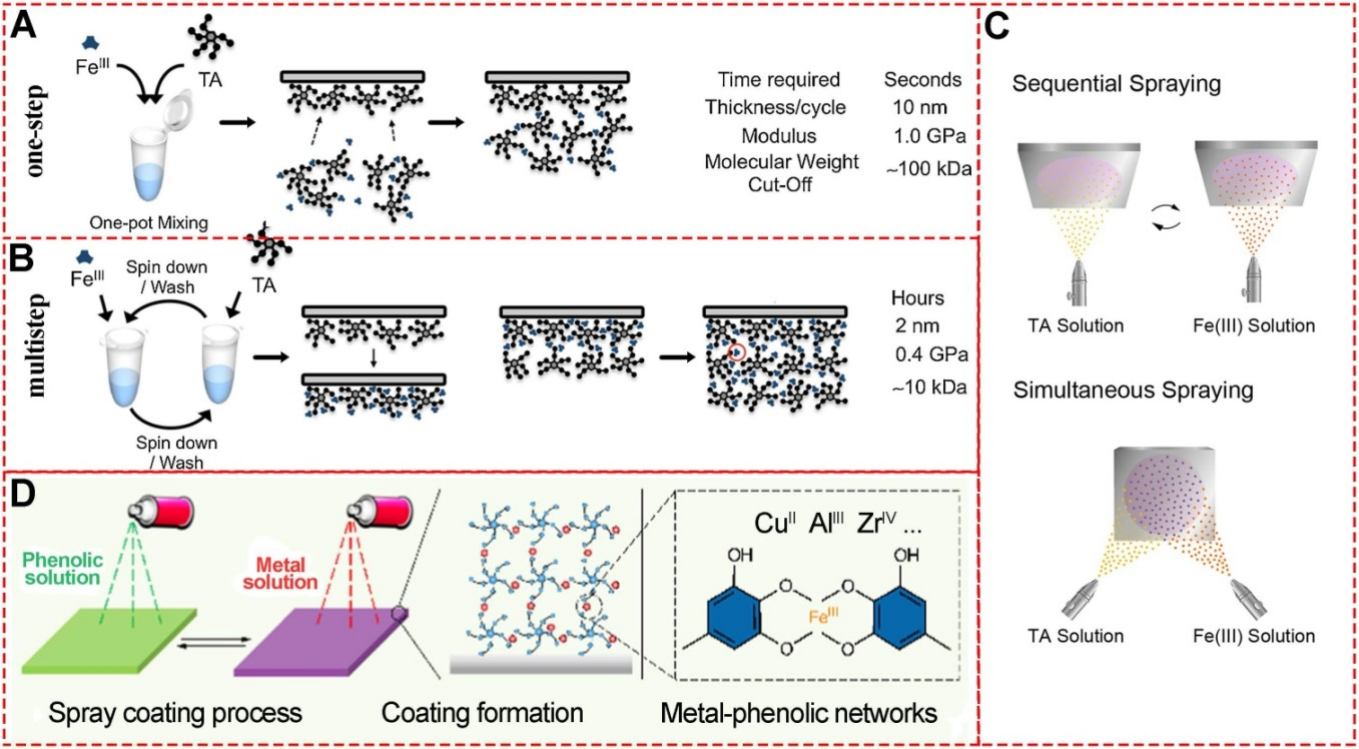
Schematic illustration of the MPN preparation through one-step and multistep assembly. (A) One-step assembly of Fe^III^ and TA. Adapted with permission from 15, copyright 2014 Angewandte Chemie International Edition. (B) Multi-step assembly of Fe^III^ and TA. Adapted with permission from 15, copyright 2014 Angewandte Chemie International Edition. (C) Fe^III^-TA spray coating and its thickness. Adapted with permission from 29, copyright 2017 Springer Nature Limited. (D) Fe^III^-TA spray coating on Quartz slides and silicon wafers. Adapted with permission from 30, copyright 2018 American Chemical Society.

The development of versatile and facile strategies for MPN engineering is of significant interest, especially the strategies for easily assembling MPNs on designed substrates with proper composition, structure, shape, and size. In 2013, the “one-step” assembly of MPNs consisting of Fe^III^ and TA was first reported by Caruso and colleagues 8. In this system, the MPN film deposition on particulate polystyrene templates occurs upon mixing the organic ligand (TA) and the inorganic cross-linker (Fe^III^) in the water at ambient temperature. The original materials are readily available and inexpensive, and importantly, no other special equipment and devices are needed. During the one-step assembly of MPNs, the color of the mixture turns immediately blue upon addition of Fe^III^ and TA, and small Fe^III^-TA networks form in the suspension and subsequently bind to the surface of the polystyrene substrate (**Figure 5A**).

Besides “one-step” assembly, “multistep” routes via coordination interactions are the most used procedures to prepare MPNs. In the “one-step” assembly, phenolic ligands and metal ions are mixed in one pot, or the substrate is immersed in separate phenolic ligand or metal ion solutions. However, “multistep” assembly has unique MPN performances as the substrates are sequentially incubated in separate solutions of excess phenolic ligands or metal ions. Since the unabsorbed Fe^III^ and TA are removed during the incubation steps of “multistep” assembly, the formation of small Fe^III^-TA networks in the solution could be precisely controlled (**Figure 5B**) 15. The quartz crystal microbalance and UV-vis spectrophotometry could be used to monitor the multistep build-up of MPNs. The different assembly routes substantially influence the coordination mode and properties of final MPN products. It has been demonstrated that the MPNs synthesized through multistep assembly contain a higher Fe^III^ content (50 mol%) than those prepared through a one-step route (25 mol%). However, the one-step assembly strategy results in a higher Young's modulus 26.

MPNs have a highly ordered network structure that allows layer-by-layer spray nanocoating. This “multistep” assembly strategy of MPNs for functionalization on various substrates has been widely studied 27-29. Compared to conventional immersive nanocoating, spray nanocoating is more time-efficient and the physicochemical properties of the coating can be easily controlled. Choi *et al.*demonstrated rapid spraying of Fe^III^-TA-MOC nanofilms on gold-coated silicon wafer substrates (**Figure 5C**) 29. During the sequential spraying, TA was first sprayed for facile priming followed by Fe^III^ solution for only 5 seconds. Alternatively, TA and Fe^III^ solutions could be sprayed simultaneously to save time. Ellipsometric measurements revealed that the film thickness of MPN nanocoating was linear with spraying cycle, time and concentration. This rapid and simple spraying to form stable and uniform films on bulk substrates is suitable for application in agricultural technology. Similarly, Zhong *et al.*established a spray assembly method for patterned and homogenous MPNs by using appropriate conditions and building blocks (**Figure 5D**) 30. By adjusting the solvent, pH, concentration, and ligand-to-metal molar ratio, physicochemical properties including thickness, roughness, and dominant coordination states could be precisely determined, providing new insights into the chemistry of MPNs interfacial assembly.

**Figure 6:**
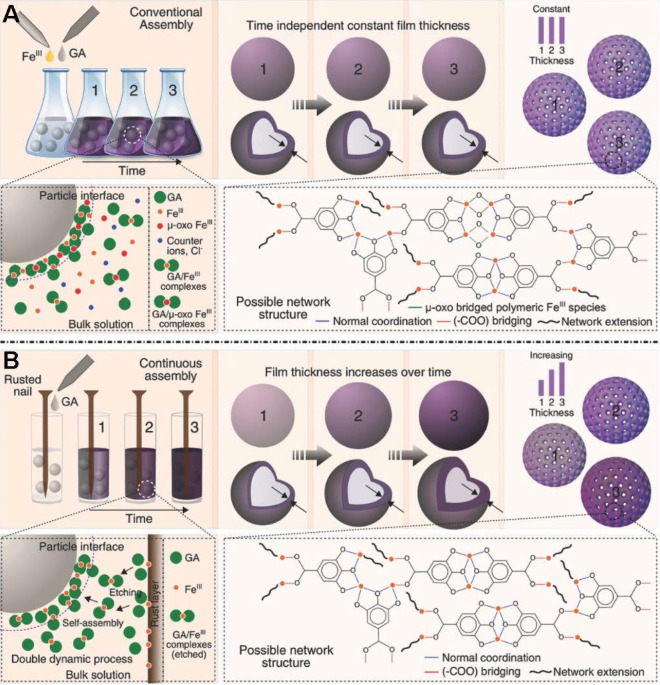
Novel, rust-mediated continuous MPN assembly process compared with the conventional assembly. Adapted with permission from 31, copyright 2017 Wiley Online Library.

The original preparation method for MPNs in the presence of solid substrates is inherently limited by the apparent discrete nature of the self-assembly procedure. Therefore, novel synthetic methods enabling continuous assembly of MPNs are required to improve processing flexibility and tunable properties. Caruso *et al.*designed a new approach using rusted iron objects as solid-state iron sources for the continuous preparation of MPNs (**Figure 6**) 31. Compared to the conventional one-step assembly, the continuous assembly process enables film thickness control as a function of reaction time. Moreover, the research work demonstrated a new concept of MPNs at the interface of materials science and green chemistry.

**Figure 7:**
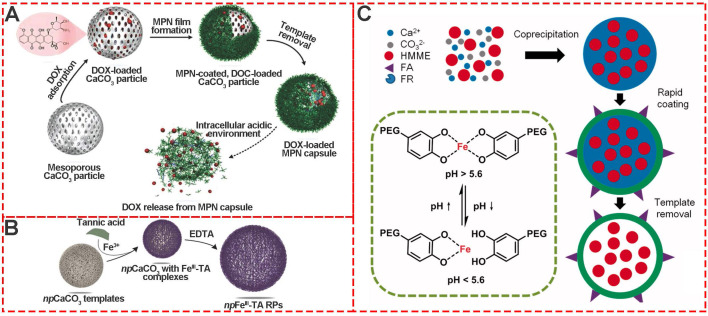
Template-mediated preparation of MPNs. (A) Schematic illustration of the fabrication process of DOX-loaded MPN capsules and release mechanism of DOX from MPN capsules. Adapted with permission from 33, copyright 2015 Wiley Online Library. (B) The formation of npFe^III^-TA RPs by replication of npCaCO_3_ template particles loaded with Fe^III^-TA complexes. Adapted with permission from 35, copyright 2015 Wiley Online Library. (C) Schematic illustration of pH-sensitive FA, HMME-doped PEG-MPN capsules for targeted photodynamic therapy against cancer cells. Adapted with permission from 32, copyright 2017 Informa UK Limited.

Template-mediated preparation is a well-known strategy for organic and inorganic nanoparticles. Calcium carbonate (CaCO_3_) particles are one of the most extensively used templates for preparing MPNs because of easy synthesis, size and shape control, and mild conditions for removing template^21^,^32^-^35^. Caruso et al. first prepared poly (styrene sulfonate) (PSS)-doped calcium carbonate (CaCO_3_) by the co-precipitation method. Subsquently, the stable Al^III^-TA self-assembly coordination films were formed *in situ* around the CaCO_3_ templates by vortexing Al^III^ ions and TA in the buffer solution (pH 8.0) (**Figure 7A**) 33. After removal of the template, the final monodispersed pH-responsive MPN capsules were obtained. Inspired by Caruso's work, Liu and coworkers designed hematoporphyrin monomethyl ether (HMME)-doped PEG-MPN capsules (MPN@HMMEs) based on monodispersed HMME-doped CaCO_3_ templates (**Figure 7C**) 32. Similarly, Feng *et al.*reported pH-responsive nanocarriers (DOX/GA-Fe@CaCO_3_-PEG) based on CaCO_3_ to enable effective intratumoral penetration and reversal of MDR 36. The Ca^II^ and Fe^II^ content in GA-Fe@CaCO_3_ was time-dependent with the extension of reaction time. These studies depicted a powerful strategy for the preparation of MPNs via template sacrifice.

**Figure 8:**
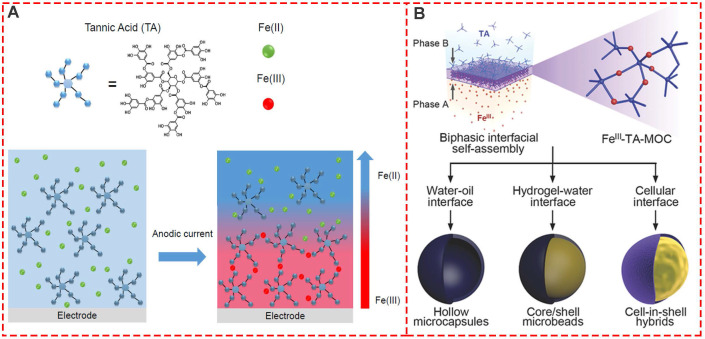
Interfacial supramolecular self-assembly for MPNs. (A) One-pot self-assembly based on the electrooxidation of Fe^II^ in Fe^III^. Adapted with permission from 38, copyright 2017 American Chemical Society. (B) Biphasic interfacial supramolecular self-assembly of Fe^III^ and TA at various interfaces. Adapted with permission from 37, copyright 2017 Wiley Online Library.

Interfacial supramolecular self-assembly for MPNs proved to be simple, fast, and effective with a high degree of uniformity and was introduced to prepare the supramolecular coordination compound of TA and Fe^III^ film based on the biphasic interfacial interaction 37. In this context, a new electro-triggered morphogenic self-assembly of Fe^III^-TA nanocoating with tunable physicochemical properties was introduced (**Figure 8A**) 38. The application of an anodic current allowed oxidation of Fe^II^ into Fe^III^, forming a confined gradient in the vicinity of the electrode and inducing localized self-assembly of a Fe^III^-TA film. The electro-triggered self-assembly of Fe^III^-TA confined Fe^III^ ion buildup on the surface of the electrode, finetuning the coating thickness by switching on and off the electrical stimulus. Previous biphasic interfacial reactions required a cumbersome procedure for synthetic molecules. Kim *et al.*first reported biocompatible supramolecular coordination complexes through biphasic interfacial reaction (**Figure 8B**) 37. Fe^III^-TA nanofilm strategy does not need complicated operating procedures, and the obtained products are biocompatible and even edible, providing great potential in the cell nanoencapsulation. Thus, the Fe^III^-TA networks synthesized in the biphasic interfacial system show high versatilities and can be seamlessly applied to various interfaces.

## Shape-controllable preparation of MPNs

**Figure 9:**
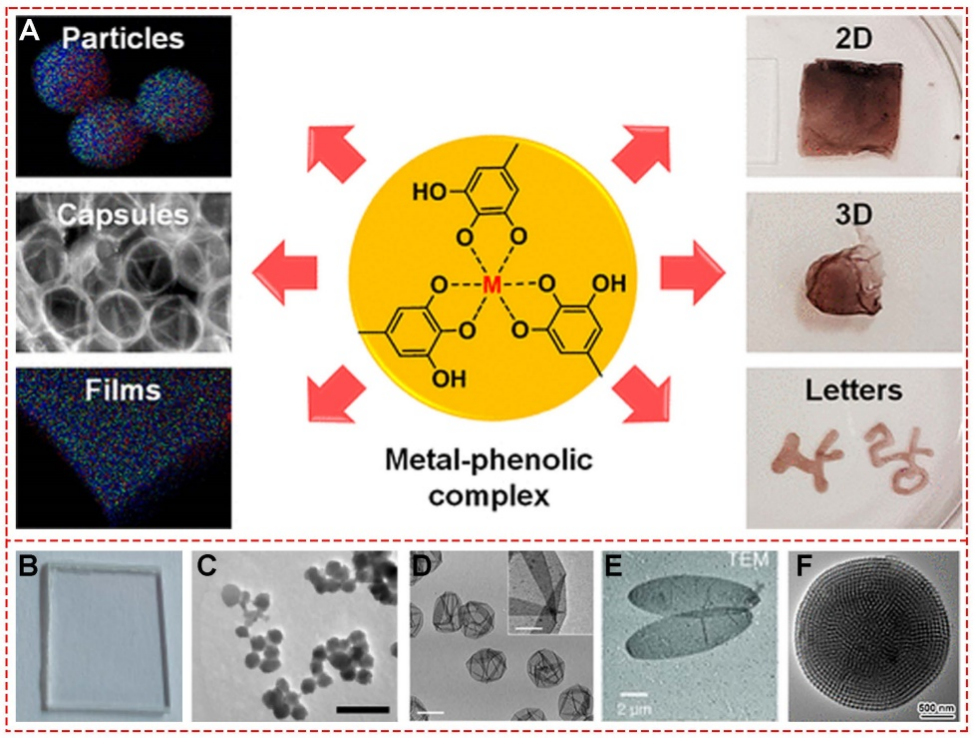
Various shapes of MPNs. (A) Schematic illustration of Fe^III^-TA networks forming different shapes. (B) Films. (C) Nanoparticles. (D) Capsules. (E) Ellipsoidal particles. (F) Mesoporous particles. (A) Adapted with permission from 39, copyright 2018 American Chemical Society. (B, E) Adapted with permission from 8, copyright 2013 American Association for the Advancement of Science. C) Adapted with permission from 40, copyright 2018 American Chemical Society. (D) Adapted with permission from 25, copyright 2014 Wiley Online Library. F) Adapted with permission from 41, copyright 2019 American Chemical Society.

In recent years, besides the discovery of novel MPN systems for new functions based on different metal ions and special phenolic or polyphenolic ligands, shape- and size- controlled preparations of 0-, 2-, and 3-dimensional MPNs also attracted much attention. Various shapes of nanoparticles, mesoporous particles, capsules, ellipsoidal particles, films, and 2D-/3D- hydrogels have been successfully fabricated with precisely controlled methods. The first and most common strategy used a well-defined template to realize the designed shape control. In 2013, Caruso first reported the one-step assembly method to prepare various films and particles 8. Subsequently, molecular self-assembly of metal ions together with various phenolic ligands was reported to form nano- to macroscopic free-standing materials with different 2D and 3D geometries (**Figure 9A**) 39. Polystyrene (PS) served as the template and TA and Fe^III^ were used as raw materials for the template-mediated preparation. The investigators deposited Fe^III^-TA films on planar PS substrates (**Figure 9B**) after completing the self-assembly coordination chemistry reaction. When spherical PS particles were used as a template, highly uniform Fe^III^-TA microcapsules were obtained after template removal (**Figure 9D**) 25,26. Chen and colleagues designed photoacoustic imaging-guided chemo-/photothermal therapy nanoparticles based on MIL-100, consisting of trimesic acid and Fe^III^ (**Figure 9C**) 40. The nanoparticle size could be optimized by changing the molar ratio of FeCl_3_·6H_2_O and 1,3,5-benzene tricarboxylic acid. The shape changed from spherical to ellipsoidal state during stretching of the spherical PS nanoparticles above their glass transition temperature. Thus, the ellipsoidal capsules of MPNs could be synthesized by sacrificing the ellipsoidal PS particles (**Figure 9E**). Recently, highly ordered mesoporous metal-phenolic particles were prepared by sacrificial double cubic network polymer cubosomes (**Figure 9F**) 41. The obtained mesoporous structure exhibited high loading efficiency of various proteins, indicating a great potential for the delivery system.

**Figure 10:**
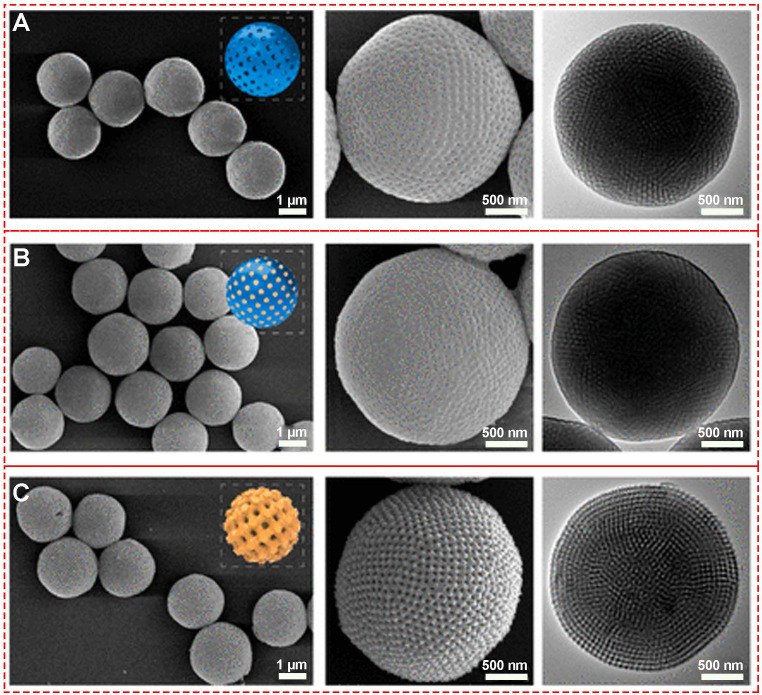
SEM and TEM images of (A) PCs, (B) PCs@MPN and (C) Meso-MPN particles. Adapted with permission from 41, copyright 2019 American Chemical Society.

Precisely controlling the shape of nanomaterials by adjusting the synthesis parameters is significant as it usually creates unique and required functions. Efforts have been made to generate mesoporous holes within the MPNs through coordinating with different ligands 42 and etching 43 and template sacrifice strategies 41., Lin et al. fabricated various mesoporous MPNs with a highly ordered single cubic network, using different metal ions (Cu^II^, Zr^IV^, Fe^III^) and phenolic ligands (gallic acid, (-)-epigallocatechin gallate) (**Figure 10**). The average diameter of the MPN-coated PCs was about 2.4 ± 0.7 μm with uniform tetragonally distributed pores of ~40 nm.

**Figure 11:**
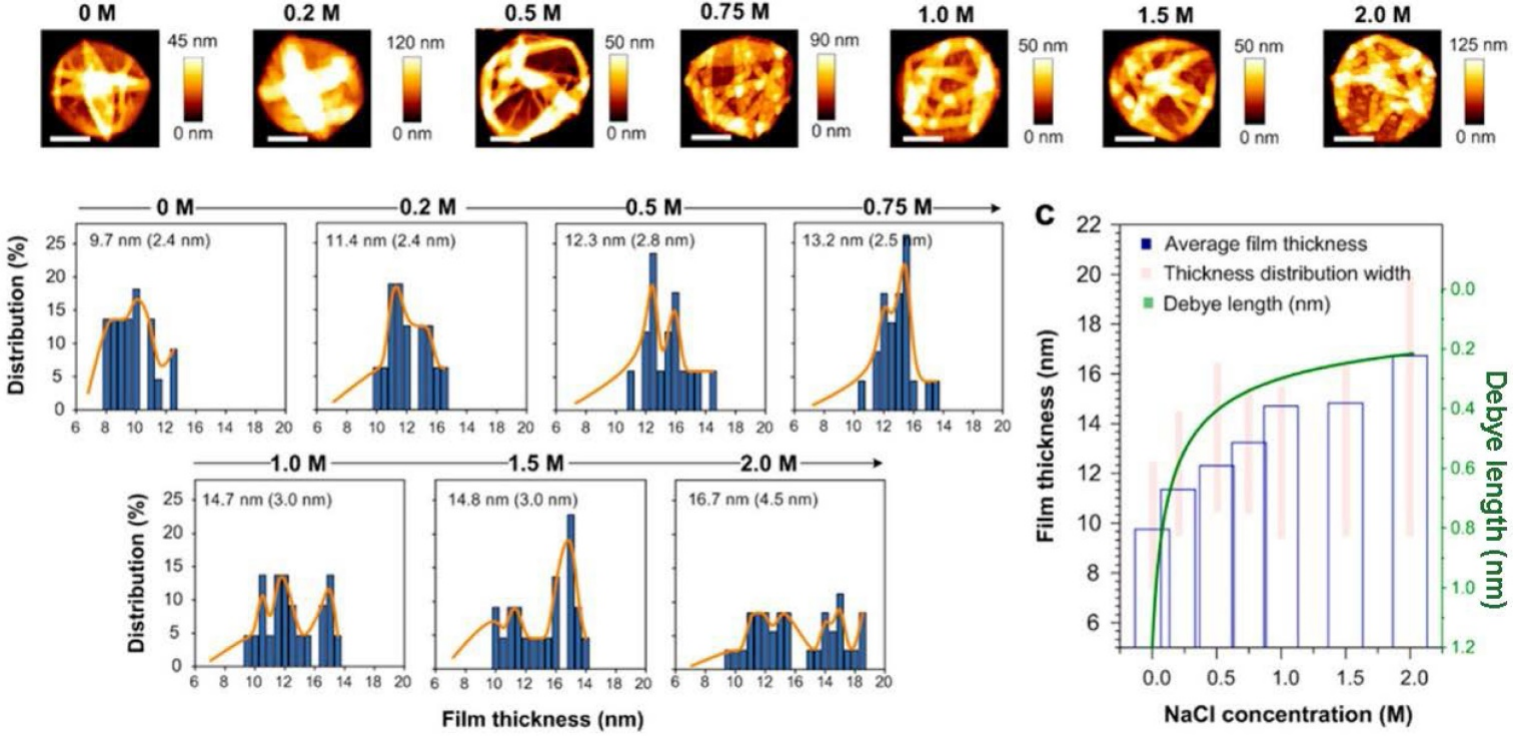
Influence of salt concentration on Fe^III^-TA film thickness. Adapted with permission from 44, copyright 2017 American Chemical Society.

The environmental conditions during the synthetic procedures are also adjusted for various shape-control. For example, Guo *et al.*systematically explored the influence of ionic strength on Fe^III^-TA MPN precursors and the final morphology and capsule thickness of MPN films 44. The results revealed that the thickness and roughness of MPN films changed under increasing 0 to 1 M NaCl concentration leading to rougher and thicker films (**Figure 11**). The molecular dynamics simulations demonstrated that the galloyl groups of Fe^III^-TA_3_ MPNs could extend further into the bulk solution away from the metal center at higher ionic strengths. This observation led to subsequent chelation with other Fe^III^ ions and/or complexes, resulting in thicker and rougher films.

## Biocompatibility of MPNs

First discovered by Caruso in 2013, MPNs are being increasingly used in nanomedical science for fabricating multi-functional nanomaterials in various applications, including tumor imaging, drug delivery systems for chemotherapy, photo-thermal therapy, radiotherapy, chemo-dynamic therapy and photo-dynamic therapy. Therefore, understanding the *in vitro* cytotoxicity and *in vivo* biocompatibility of MPNs in biological environment is of great significance.

**Figure 12:**
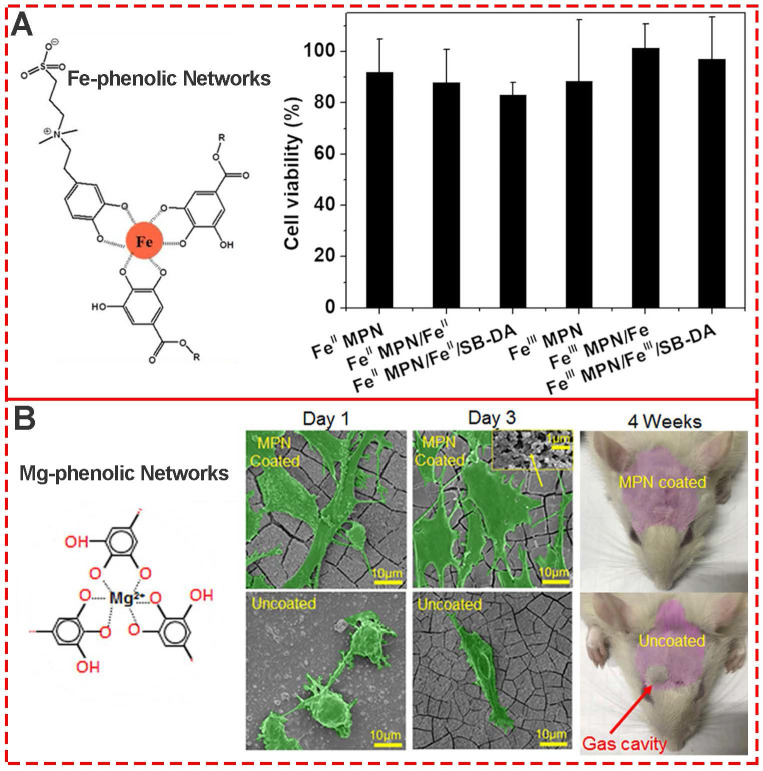
(A) Cytotoxicity evaluation of various Fe-phenolic network composites to NIH-3T3 fibroblasts. Adapted with permission from 45, copyright 2020 Elsevier B.V. (B) Morphology assessment of MC3T3-E1 cells seeded on Mg-phenolic networks for 1 and 3 days. Adapted with permission from 46, copyright 2019 American Chemical Society.

As one of the most common components of MPNs, iron ions and TA are generally recognized as safe (GRAS) by the U.S. Food and Drug Administration, and Fe^III^-TA networks have generated much interest for various biomedical applications. To evaluate the biocompatibility of both Fe^II^ and Fe^III^ MPNs, Huang *et al.*used NIH-3T3 fibroblasts to systematically evaluate the cytotoxicity of all MPN coatings 45. The MTT assay showed that cells co-incubated with substrates with different MPN coatings were above 80% viable (**Figure 12A**), indicating negligible cytotoxicity and good biocompatibility. Besides the relative cell viability, Li et al. explored the change in morphology of cells cultured on various MPN surfaces (**Figure 12B**) 46. After 3 days of culture, MC3T3-E1 cells attached well to the raw and 1.2%Mg@TA, 2.4%Mg@TA, 3.6%Mg@TA, and NaOH-treated samples, indicating the suitability of surfaces for MC3T3-E1 cell proliferation. Moreover, with the increased concentration of Mg in Mg@TA networks, better adhesion and proliferation of the bonelike cells were observed, indicating the osteocompatibility of Mg-phenolic coating.

**Figure 13:**
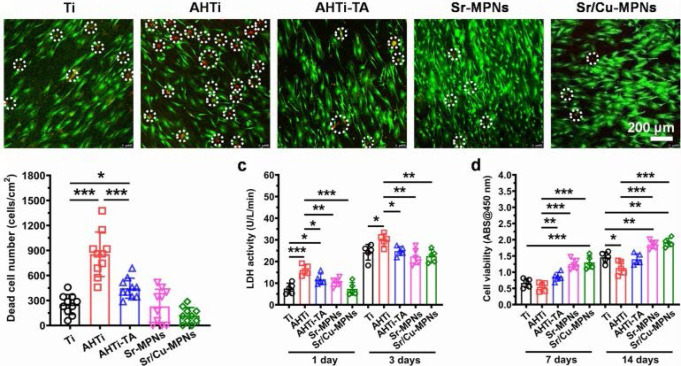
Cell compatibility evaluation of five Ti samples. Adapted with permission from 47, copyright 2020 Elsevier B.V.

Similarly, Cai et al. prepared MPNs on pure Titanium (Ti) substrates to form Ti-based implants and explore their biocompatibility (Ti, AHTi, AHTi-TA, Sr-MPNs, and Sr/Cu-MPNs) 47. Compared to the substrates without TA or MPNs, fewer dead cells were observed on the Ti- based substrates, indicating that TA and MPNs could significantly improve the biocompatibility of the substrates (**Figure 13**). Furthermore, the mesenchymal stem cells grown on the MPN-coated Ti implants showed superior adhesion and the highest cell viability after 7 days of seeding compared with the bare Ti substrates. The enhanced biocompatibility of TA or MPN coatings might originate from the free radical scavenging capability of phenolic hydroxyl in phenolic molecules. Also, the biocompatibility assays of Fe^III^-based MPN hydrogels indicated that the potential cytotoxicity of some metal ions is tempered by the strong coordination between metal ions and phenolic ligands 48.

**Figure 14:**
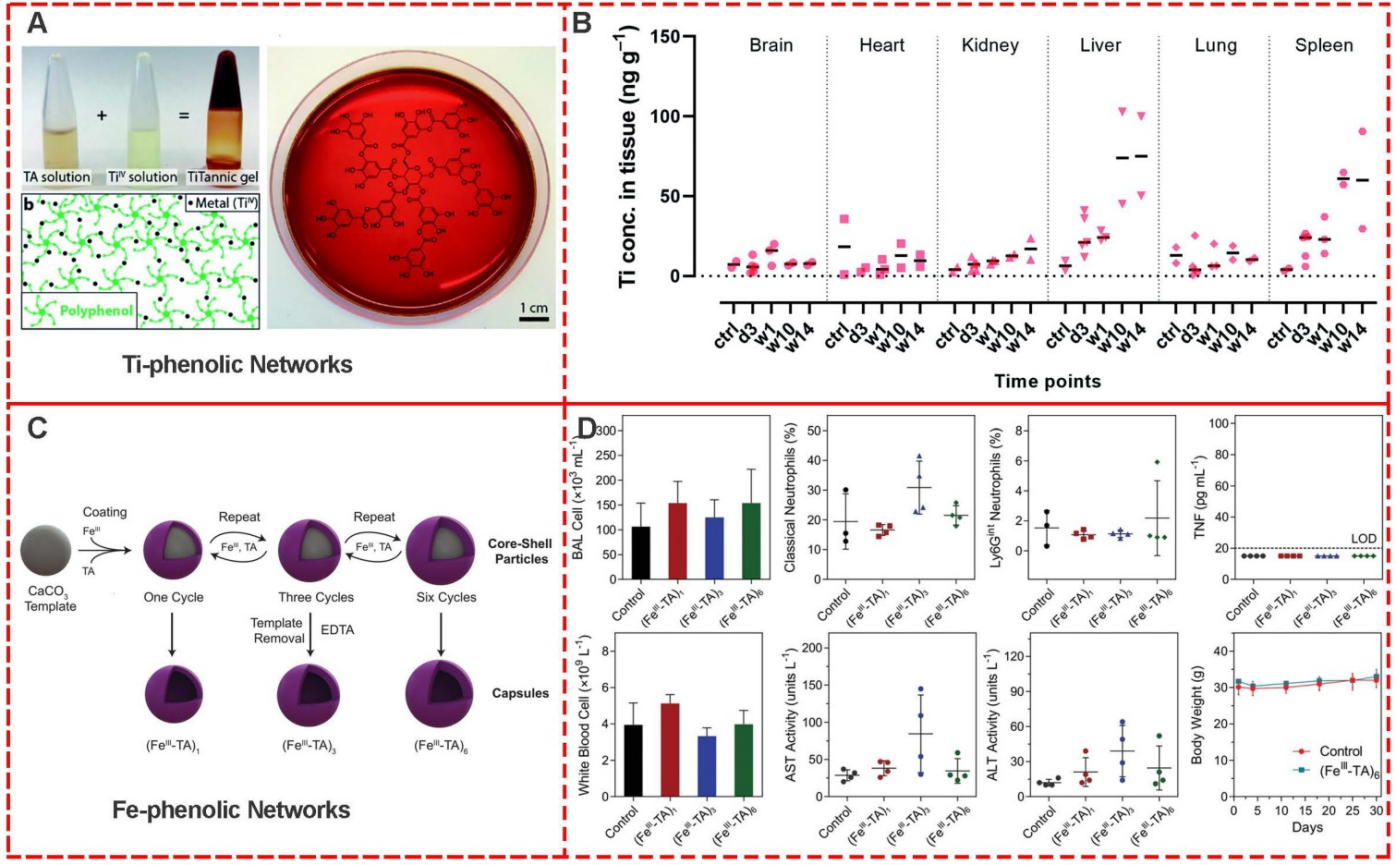
(A, B) Preparation scheme and bio-distribution studies of Ti-Tannic hydrogel. Adapted with permission from 50, copyright 2019 Royal Society of Chemistry. (C, D) Preparation scheme and *in vivo* assessment of inflammation and toxicity of the Fe-based phenolic capsules. Adapted with permission from 52, copyright 2020 Wiley Online Library.

The assessment of *in vivo* biocompatibility of a material is a mandatory prerequisite for its clinical use, requiring well-planned preclinical and clinical trials 49. Stevens *et al.* systematically evaluated the *in vivo* biocompatibility and immunogenicity of the metal-phenolic gelation for 14 weeks 50. Compared to the blank control group, there were no statistical differences in Ti concentration in the brain, heart, and kidney of mice injected with below 50 ng [Ti]-Tannic gels per g of tissue , indicating their safty for normal tissues (**Figure 14A**) 51. In spleen and liver tissues of mice, an increased concentration of Ti was observed with 50-100 ng Ti per g of tissue after 14 weeks (**Figure 14B**). The overall low Ti concentration in main organs including the brain, heart, kidney, liver, lung, and spleen exhibited excellent *in vivo* biocompatibility of metal-phenolic gelation.

Caruso et al. investigated the *in vivo* toxicity and *in vivo* inflammatory response of nebulized metal-phenolic capsules in mice 52. Fe^III^-TA capsules with different thicknesses (108 nm for (Fe^III^-TA)_1_, 162 nm for (Fe^III^-TA)_3_, 207 nm for (Fe^III^-TA)_6_) were systematically examined, including the serum levels of liver enzymes and proteins. The major hematological parameters, total number of cells and levels of inflammatory cytokines in the BAL fluid, and lung histology after 4 and 24 h post-administration were examined (**Figure 14C and 14D**). After 30 days' administration with (Fe^III^-TA)_1_, 162 (Fe^III^-TA)_3_, and (Fe^III^-TA)_6_, no signs of lethargy, higher levels of inflammatory cytokines, higher BAL cell numbers, lung inflammation, or alveolar damage were observed compared with the control group. Furthermore, hematological parameters showed no significant changes of liver enzymes and protein levels. These results indicated high *in vivo* biocompatibility of Fe^III^-TA capsules.

In 2015, Wang and colleagues demonstrated a gram-scale preparation strategy to synthesize a class of renal-clearable nanodots (Fe-CPNDs) via the self-assembly coordination of Fe^III^, GA and PVP 53. The *in vivo* biodistribution investigation exhibited that Fe-CPND excretion from the body after intravenous injection was via renal clearance. Pharmacokinetic measurements of Fe-CPNDs in blood showed that the blood-elimination half-time was about 5.5±1.9 h. No significant differences were observed between Ge-CPNDs-treated and control groups during 30 days of treatment by histochemical, hematological, and blood biochemical analyses, confirming the nontoxicity of Fe-CPNDs. Although appropriate MPN concentrations were biocompatible *in vivo,* extensive *in vitro* and preclinical studies are required before future clinical applications of MPNs.

## Cancer theranostic applications of MPNs

Theranostics provides a transition from conventional medicine to personalized medicine and has emerged as a safe, targeted, and efficient pharmacotherapy 54,55. Advances in nanotechnology have furnished multi-functional nano-platforms, enabling multimodal imaging with a combination of two or more therapeutic modalities for simultaneous therapy and diagnostics 56,57. Among these nano-platforms, MPNs show unique performances and an excellent integrated capacity via controlling the physicochemical properties for biological and biomedical applications, such as tumor imaging, drug delivery system, radiotherapy, photo-thermal therapy, and chemo- and photo-dynamic therapy 7. Here, we provide an overview of the existing knowledge and current update of engineering MPNs for cancer theranostics.

## Platforms for tumor imaging

Molecular imaging of tumors has generated wide attention for its ability to interrogate and diagnose tumor tissues without biopsies or surgical procedures 58. The information obtained from molecular imaging is applied to evaluate the disease state, monitor treatment, and assess therapeutic effects. Imaging plays an essential role in all phases of cancer management including screen, prediction, detection, staging, prognosis, therapeutic planning, therapy response, recurrence, and palliation 34,59.

**Figure 15:**
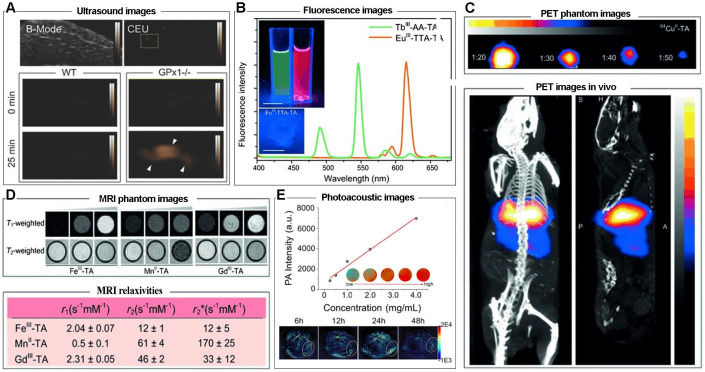
(A) Representative brightness mode (B-mode) and contrast-enhanced ultrasound (CEU) images of mouse livers over time *ex vivo*. Adapted with permission from 35, copyright 2015 Wiley Online Library. (B) Fluorescence spectra and images of capsule suspensions of Eu^III^-TTA-TA and Tb^III^-AA-TA excited at 360 nm. (C) Pseudo-color PET phantom and *in vivo* small-animal PET/CT image of 64 Cu^II^-TA capsule suspensions at different dilution ratios. (D) MRI phantom images and MRI relaxivities of Fe^III^-TA, Mn^II^-TA, and Gd^III^-TA capsules immobilized in agarose. (B, C, D) Adapted with permission from 25, copyright 2014 Wiley Online Library. (E) PA intensity of MCH NP solutions and PA images of MCH NP both *in vitro* and *in vivo*. Adapted with permission from 40, copyright 2018 American Chemical Society.

Due to the intrinsic performance of metal ions and the porous network structure, MPNs are excellent candidates as imaging agents or carriers. For example, nanoporous metal-phenolic particles (*np*Fe^III^-TA RPs) were fabricated via the self-assembly coordination complexation of Fe^III^ ions and TA, and the nanoporous CaCO_3_ particles. The Fe^III^ chelated in the *np*Fe^III^-TA RP networks could catalyze H_2_O_2_ into O_2_ and act as an agent for ultrasound imaging (**Figure 15A**). Also, Tb^III^-TA and Eu^III^-TA capsules have been shown to be excellent fluorescence imaging agents; acetylacetone (AA) and 2-thenoyltrifluoroacetone (TTA) were employed as co-ligands to impart fluorescence intensities of Tb^III^-TA and Eu^III^-TA complexes, respectively. Due to the ^5^D_0_-^7^F_2_ transition, Tb^III^-AA-TA exhibited green fluorescence around 545 nm and Eu^III^-TTA-TA red fluorescence around 613 nm (**Figure 15B**) 25. Furthermore, due to the functionalities of metal ions in phenolic networks, MPNs could act as agents for positron emission tomography (PET). ^64^Cu^II^-TA capsules were fabricated by doping ^64^Cu in the MPNs. PET phantom images and PET images *in vivo* in healthy BALB/c mice suggested that ^64^Cu^II^-TA capsules were efficient PET-active agents, and could be used to track the bio-distribution of theranostic platforms or drugs (**Figure 15C**).

Gd^III^, Fe^III^, and Mn^II^ are frequently used as raw materials for preparing imaging nano-platforms for MRI contrast agents, and Gd^III^-TA, Fe^III^-TA, Mn^II^-TA had *T*_1_-weighted and *T*_2_-weighted MRI properties, with *r*_1_ value of 2.31, 2.04, 0.5 s^-1^ mM^-1^ and *r*_2_ value of 46, 12, 61 s^-1^ mM^-1^, respectively (**Figure 15D**). Mn^II^-TA capsules exhibited the greatest MRI performance with relaxivity of 60 s^-1^ mM^-1^, which is generally sufficient for *in vivo* use 60. As mentioned above, dopamine (PDA) is one of the functional coordination ligands for MPNs. By introducing PDA and curcumin in MPNs, a photoacoustic imaging (PAI)-guided chemo-/photothermal combination therapeutic agent was obtained (**Figure 15E**) 40. *In vivo* PAI measurement in HeLa cells tumor-bearing mice showed an imaging signal peak at 24 h post-injection, suggesting the considerable PAI ability of MPNs. These results demonstrated that MPNs are a potential platform for tumor imaging due to the excellent encapsulation capacity for loading imaging agents and the intrinsic performance of tumor imaging.

## Drug delivery systems for chemotherapy

**Figure 16:**
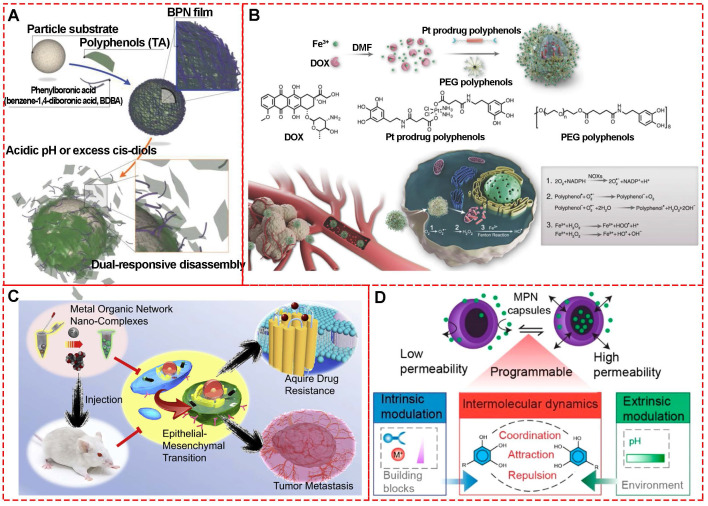
(A) Formation and dual-responsive disassembly of a BPN film for controlled anticancer drug delivery. Adapted with permission from 66, copyright 2015 Wiley Online Library. (B) Formulation of nanoparticles and the ROS enhanced chemotherapy mechanism of lipid oxidation and DNA damage to enhance combination chemotherapy. Adapted with permission from 67, copyright 2018 Wiley Online Library. (C) Schematic diagram of the EIN-coated nanomedicines to prevent cancer cells from gaining drug resistance and eliminate EMT-type cancer cells for inhibiting tumor metastasis. Adapted with permission from 69, copyright 2017 Elsevier B.V. (D) Schematic of the assembly of phenolic ligands and metal ions to form an MPN film on a PS template particle. Adapted with permission from 70, copyright 2020 American Chemical Society.

Approaches for delivering therapeutic agents to tumor sites typically include systemic administration of therapeutic drug delivery systems or localized delivery of therapeutics to the target tissue 61. Generally, encapsulation of chemotherapeutic drugs in nanocarriers could improve the solubility, biocompatibility, bioavailability, bio-distribution, and targeting. However, only about 0.7% of nanocarriers could be successfully delivered to target solid tumors 62. Therefore, it is imperative to explore various approaches to increase the efficiency of drug delivery systems, including designing novel nanocarriers, altering physicochemical performances, or using biological techniques 63, 64. MPNs, especially with the hollow capsule structure, have attracted great interest as drug carriers due to their appropriate physicochemical and biomedical properties, including broad raw materials, elective permeability, suitable size, excellent biocompatibility, and external stimuli-responsive functionalities 65. For example, a biologically relevant boronate-phenolic network with dual-responsive performances was fabricated by complexing phenolic materials and phenylborate (**Figure 16A**) 66. The *cis*-diol responsiveness of boronate complexes and pH responsiveness of MPNs were brought together by the dynamic boronate covalent bonding of reversible boronate ester based on the multivalent coordination chemistry. Due to the controlled release of the boronate-phenolic network, the DOX release from networks could be dramatically accelerated when the pH decreased from 7.4 to 5.0 or 100 × 10^3^ M mannitol was added. Furthermore, the combination of acidic pH and *cis*-diols could further accelerate the release kinetics of DOX from the networks due to the synergistic effect between the dual-responsive strategies.

Similarly, Chen et al. developed a ROS-promoting combination drug delivery system (DOX@Pt prodrug Fe^III^ nanoparticles, DPPF NPs) via a self-assembly process based on Fe^III^-MPNs for co-delivery of platinum prodrugs and DOX 67. Both co-delivered compounds could activate nicotinamide adenine dinucleotide phosphate oxidases and generate superoxide radicals (*O*_2_^.-^) to kill tumors (**Figure 16B**). The polyphenols in DPPF NPs could catalyze 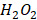 generation from (*O*_2_^.-^) due to their superoxide dismutase-like activity, and the *·OH* generated by Fenton reaction could improve the chemotherapy efficiency via a cascade of synergistic bioreactions. *In vivo* antitumor evaluation based on the U87MG xenograft tumor model exhibited highest tumor growth inhibition by DPPF NP treatment, indicating the synergistic effect of ROS-promoted chemotherapy.

It is well-established that tumor metastasis and multidrug resistance are the core challenges for cancer treatment. Emerging evidence has demonstrated that epithelial-mesenchymal transition (EMT) is associated with drug resistance and also an essential step in tumor metastasis 68. Therefore, it is imperative to eliminate EMT cells with high metastatic capability and intensified drug resistance during chemotherapy. However, EMT could be induced under the selective pressure of clinical cytotoxic drugs during chemotherapy. To solve this problem, Zhang et al. designed a multi-functional epigallocatechin gallate/iron nano-complexes (EIN) as a drug delivery system to improve conventional treatment (**Figure 16C**) 69. The *in vitro* and *in vivo* results revealed that EIN restricted tumor metastasis and also eliminated EMT-type cancer cells. The study provides a novel strategy to develop MPNs as chemotherapeutic agent carriers. Especially, MPNs exhibit selective gating performance under different pH environments to switch permeability, which is important for smart drug delivery 70. Caruso's study based on Fe^III^, Al^III^, Cu^II^ self-assembly coordination with tannic acid, gallic acid, and pyrogallol revealed that the synthesized MPN capsules could switch between “closed” and “open” states in response to the pH change between 4 and 9 (**Figure 16D**). These phenomena indicated that the designed MPNs could be precisely controlled for dynamic cargo encapsulation and release for drug delivery.

## Agents for PTT

Near-infrared (NIR) absorbing materials, namely photosensitizers, have attracted considerable interest in recent years for phototherapy of malignant tumors due to their ability to convert NIR light into heat. So far, photothermal agents, including carbon nanomaterials, metal nanoparticles, transition metal dichalcogenides, upconversion nanoparticles, black phosphorus-based complexes, and photosensitizer-based polymers are still scarce. In this context, MPNs were found to have excellent photo-thermal conversion performance 53. The mechanism could be due to the coordination effect between metal ions and polyphenol of MPNs that can induce splitting of the d orbitals of metal ions, resulting in d-d electronic transitions and appearance of a new absorption peak 71,72. The NIR absorption ability can be adjusted by manipulating the coordination ligands as the d-d electronic transitions of metal ions strongly depend on the ligand properties. Therefore, much attention has been focused on the design of novel MPNs for efficient phototherapy of tumors.

**Figure 17:**
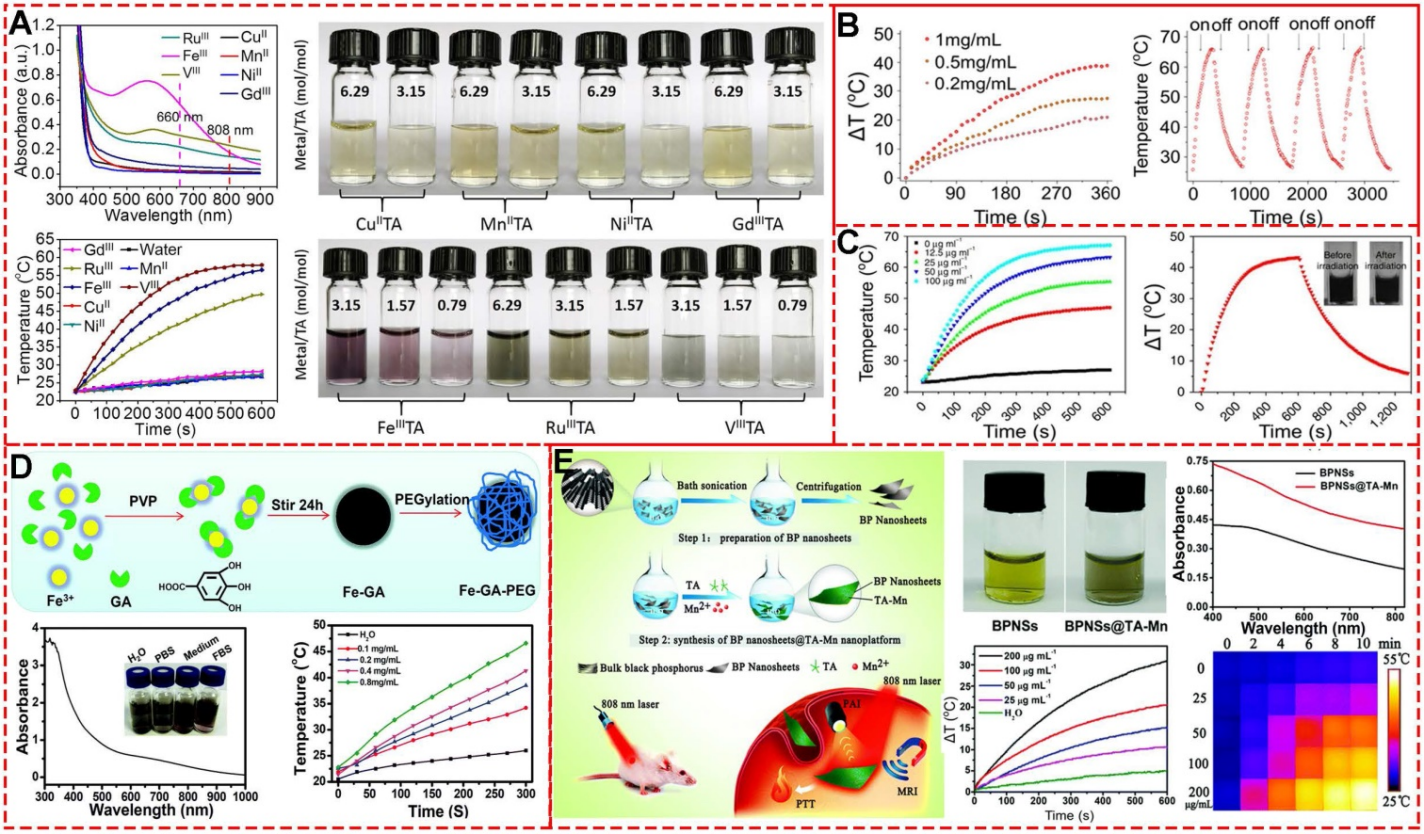
Photothermal evaluation of different MPNs. (A) Cu^II^TA, Mn^II^TA, Ni^II^TA, Gd^III^TA, Ru^III^TA, V^III^TA, and Fe^III^TA (100 μg mL^-1^, metal/TA = 3.15, 2 W cm^-2^). Adapted with permission from 12, copyright 2018 American Chemical Society. (B) Polydopamine-modified hyaluronic acid (HA-PDA)-coated MOF nanoparticles. Adapted with permission from 40, copyright 2018 American Chemical Society. (C) Poly (vinylpyrrolidone) (PVP)-protected Fe^3+^-gallic acid (GA) coordination polymer nanodots (Fe-CPNDs) under NIR 808-nm laser irradiation with 1.3 W cm^-2^ power. Adapted with permission from 53, copyright 2015 Springer Nature Limited. (D) Fe-GA-PEG CPNs in water with different concentrations under NIR 808-nm laser irradiation at 0.8 W cm^-2^ power density. Adapted with permission from 74, copyright 2017 Royal Society of Chemistry. (E) Tannic acid (TA)-Mn^2+^ chelate networks on black phosphorus (BP) nanosheets (BPNSs) (1 W cm^-2^). Adapted with permission from 75, copyright 2018 Royal Society of Chemistry.

Feng et al. systematically explored the photo-thermal capability of MPNs by inducing different metal ions, including Gd^III^, Ru^III^, Fe^III^, Cu^II^, Ni^II^, Mn^II^, and V^III^ (**Figure 17A**) 73. Under the irradiation of NIR 808-nm laser, the temperature of Fe^III^-TA nanospheres with a concentration of 200 μg mL^-1^ increased about 44.5 °C compared to 3.8 °C of water. Further measurements showed that V^III^-TA and Ru^III^-TA nanospheres exhibited considerably broad absorption in the NIR window and NIR-activated photo-thermal conversion, while Gd^III^, Cu^II^, Ni^II^, Mn^II^-based MPNs. Besides TA, pH-sensitive MPNs with excellent degradability-based on Fe^III^ and trimesic acid were combined with curcumin and PDA-modified hyaluronic acid for imaging-guided chemo-/photo-thermal treatment of tumors (**Figure 17B**) 40. The final composites displayed a notable temperature change of 25.2 °C under NIR 808-nm laser irradiation for 6 min with 1.0 W cm^-2^ with the photo-thermal conversion efficiency of about 21.0%, close to that of Au nanorods (23.7%). Furthermore, a simple and scalable procedure was performed to prepare pH-activated nanodots (Fe-CPNDs) based on the self-assembly coordination of GA, Fe^III^, and poly (vinylpyrrolidone) 53. The obtained Fe-CPDNs showed strong absorption of light, indicating the potential of photo-thermal conversion effect. After 5 min of NIR 808-nm irradiation, the temperature of Fe-CPNDs with 25 μg mL^-1^ [Fe] increased to 50 °C, enough to kill cancer cells (**Figure 17C**). Similarly, by mixing GA and Fe^III^ solutions, Cheng *et al.*prepared polyethylene glycol-modified ultra-small Fe-GA self-assembly coordination nanoparticles for phototherapy of cancer (**Figure 17D**) 72,74. The temperature-dependent nanoparticles at 0.8 mg mL^-1^ showed temperature increase by 20 °C under NIR 808-nm laser irradiation (0.8 W cm^-2^) for 5 min. The photo-thermal efficacy of the ultra-small nanoparticles was confirmed by *in vivo* photo-thermal therapy experiments in 4T1 tumor-bearing mice.

As discussed above, various metal ions have been explored for multi-functional applications. Yang et al. directly used Mn-TA networks to coat black phosphorus nanosheets and obtained Mn-TA chelated-coated BPNSs (**Figure 17E**) 75. The synthesized BPNSs exhibited multi-functional performances, including photoacoustic imaging, Mn^2+^-induced *T*_1_-weighted MRI contrast enhancement, and photo-thermal properties. As excellent photosensitizing agents and photosensitizing agent carriers, various MPNs have demonstrated their efficient tumor killing ability *in vitro* and *in vivo*. Future studies should focus on designing new MPN systems with greater abilities and clinical application potential.

## Agents for radiotherapy

Radiotherapy is one of the most common and effective clinical modalities for cancer treatment, with about more than 60% of patients receiving radiotherapy 76. Although innovative technologies have significantly improved RT efficiency, challenges like insufficient radiation dose and hypoxia-associated radio-resistance remarkably lower the radio-therapeutic efficiency and induce radiation resistance, ultimately leading to the failure of RT 77,78. Also, a high dose of radiation always results in normal tissue toxicity. Therefore, it is imperative that novel RT approaches must reduce treatment toxicity while providing sufficient therapeutic efficacy.

**Figure 18:**
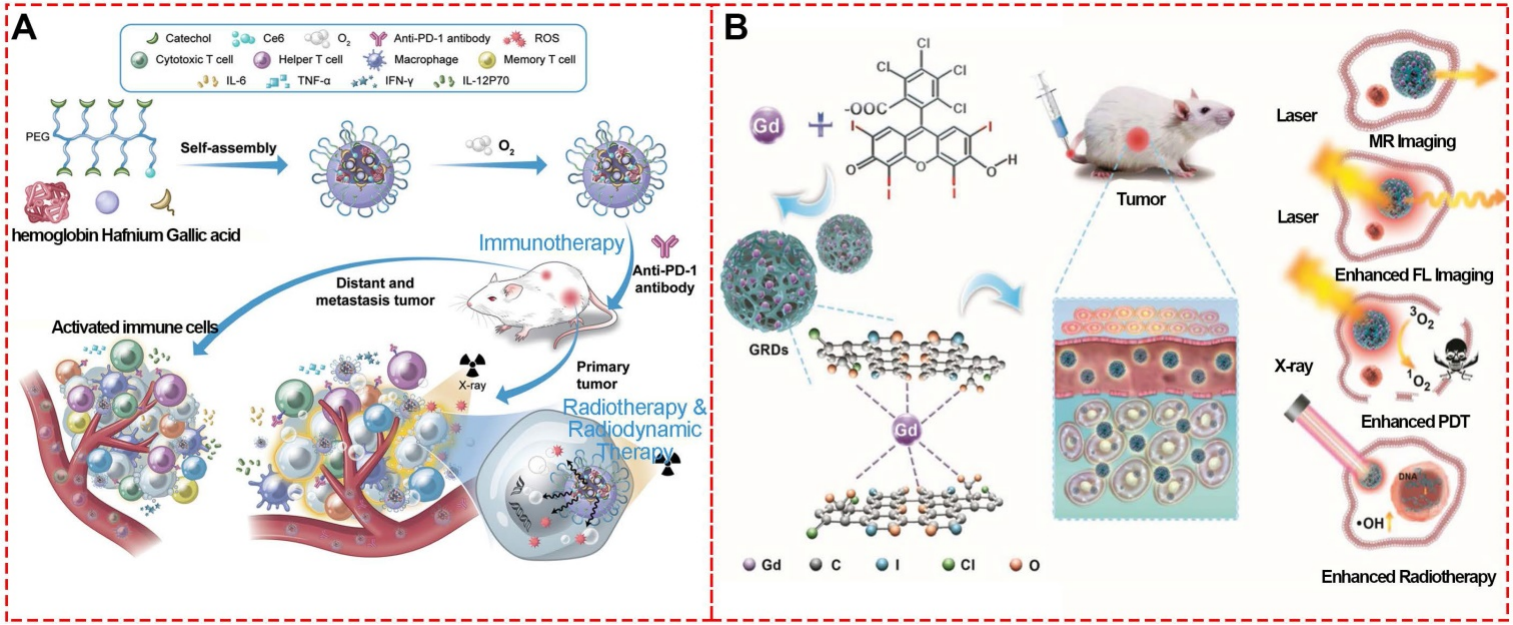
Radiotherapy based on MPNs. (A) Schematic illustration of synthesized Hb@Hf-Ce6 nanoparticles for radiotherapy, radiodynamic -immunotherapy to eradicate primary and remote tumors. Adapted with permission from 79, copyright 2020 Wiley Online Library. (B) Schematic illustration of GRD preparation and *in vivo* fluorescence-/MR-imaging-guided PDT and radiotherapy of 4T1 tumor cells using GRDs. Adapted with permission from 80, copyright 2020 Wiley Online Library.

Nanotechnology is a potential approach to address challenges, like intensity-modulated, imaging-guided, and dose-guided radiotherapy, deep inspiration breath-hold (DIBH), and combination therapy 78. To address the limitations of high dose-dependent tissue toxicity, non-selectivity, and radio-resistance, Dai and colleagues designed a facile X-ray nano-processor (Hb@Hf-Ce6 NPs) based on metal-phenolic self-assembly coordination for sufficient oxygen delivery *in vivo* and X-ray-triggered ultrasensitive ROS generation in the tumor microenvironment (TME) (**Figure 18A**) 79. In this system, hemoglobin (Hb), hafnium (Hf) and chlorin e6 (Ce6) were used for oxygen delivery, high-Z radiosensitizer, and efficient radio-luminescence, respectively. Singlet oxygen sensor green test showed that a high level of ^1^O_2_ could be generated from Hb@Hf-Ce6 NPs under irradiation of 8 Gy X-ray compared to Hb@Hf-Ce6 NPs without irradiation. Quantitative fluorescence intensity detection exhibited 7.29 times higher ^1^O_2_ level under X-ray irradiation than free Ce6.

The Hb@Hf-Ce6 NP-based X-ray irradiation was used to evaluate the *in vivo* antitumor efficacy and showed significant growth inhibition on both primary and distant tumors in a 4T1 orthotopic and bilateral tumor model. Furthermore, treatment efficacy of the combination strategy of RT-radiodynamic therapy (RDT) and PD-1 checkpoint blockade immunotherapy based on the Hb@Hf-Ce6 NPs was demonstrated in the lung metastasis model in which the experimental group of NP(+) and programmed cell death protein 1 (PD-1) (+) exhibited delayed metastasis without mouse death. These results indicated that Hb@Hf-Ce6 NPs could improve the therapeutic effect of RT-RDT, enhance modulation of hypoxia TME and promote antitumor immune response in combination with PD-1 immune checkpoint blockade.

Similarly, gadolinium (Gd)-rose Bengal (RB) coordination polymer nanodots (GRDs) based on their self-assemble coordination (**Figure 18B**) 80 exhibited a 1.9-fold increase in singlet oxygen generation efficiency and 7.7-fold luminescence intensity enhancement compared with free RB due to the self-assemble coordination of metal-phenolic structure. The *in vivo* combined PDT and RT showed 98.8% tumor inhibition with the smallest tumor sizes, while the tumor inhibition rates for PDT or RT alone groups were 50.5% and 43.8%, respectively. These properties demonstrated that GRDs could be effectively applied to MRI-/enhanced-fluorescence-imaging-guided PDT and radiotherapy for cancers.

## Agents for CDT and PDT

Since the discovery of Fenton/Fenton-like reactions triggered by the TME, chemodynamic therapy (CDT) of tumors is considered a great potential treatment with minimal side effects. The decomposition of hydrogen peroxide could be catalyzed by transition metal ions like Fe, Cu, Mn, Ni, and Co to produce hydroxyl radicals 81, damaging biomolecules, such as lipids, proteins, and nucleic acids via high oxidative stress and inducing cancer cell death. In this context, MPNs are natural candidates as chemo-dynamic agents that could easily release transition metal ions by external triggers, for example low pH in TME.

To fabricate a synergistic combination therapeutic platform based on MPNs enhanced by toxic reactive oxygen species, Fe^III^, platinum prodrug polyphenols, hydrophobic DOX, and polyethylene glycol polyphenols were chosen as raw materials to construct DOX@Pt prodrug Fe nanoparticles (DPPF NPs) 67. In this system, DOX and platinum prodrug polyphenols induced cisplatin to activate NOXs and produce O_2_^Ÿ-^, the polyphenols could act as a SOD-like enzyme to catalyze O_2_^Ÿ-^ to H_2_O_2_, and finally the ferric ions could react with H_2_O_2_ to generate highly toxic *·OH* killing cancer cells by Fenton reaction. *In vivo* evaluation using the U87MG xenograft tumor model revealed that the DPPF NPs-treated group had tumor growth inhibition and high survival rate.

**Figure 19:**
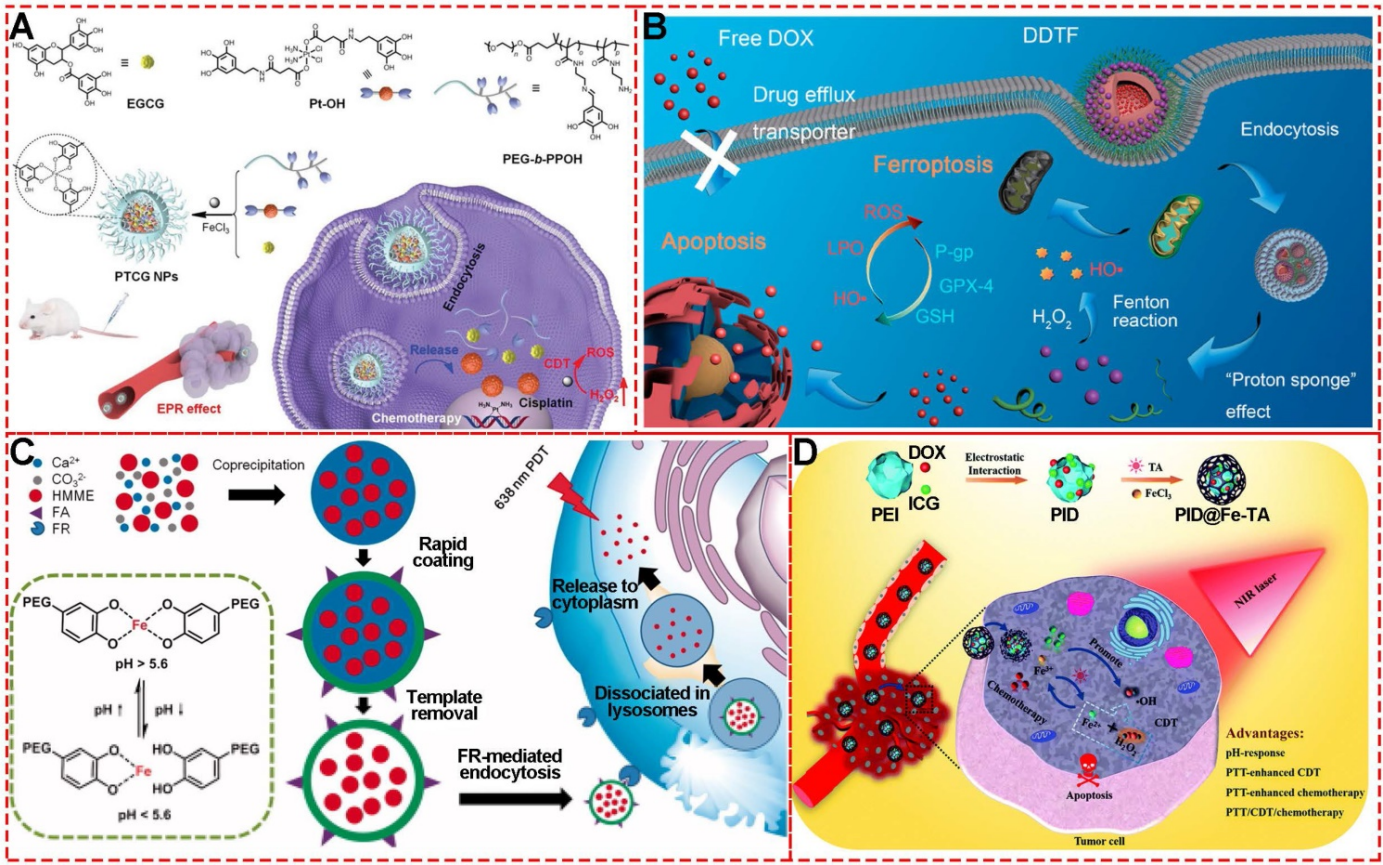
(A) Chemical structures and cartoon illustration of the building blocks (EGCG, Pt-OH, PEG-b-PPOH) used for the preparation of PTCG NPs to realize chemo/chemo-dynamic therapy. Adapted with permission from 82, copyright 2019 Wiley Online Library. (B) Schematic illustration showing the construction of DDTF nanocomplexes and their application for apoptosis/ferroptosis-mediated cancer therapy. Adapted with permission from 16, copyright 2019 American Chemical Society. (C) Schematic illustration of pH-sensitive FA and HMME-doped PEG-MPN capsules for targeted photodynamic therapy against cancer cells. Adapted with permission from 32, copyright 2017 Informa UK Limited (D) Fabrication process of PID@Fe-TA nanocomposites for cancer treatment by CDT/photo-thermal therapy/chemotherapy synergistic effect. Adapted with permission from 85, copyright 2020 Royal Society of Chemistry.

Mao et al. synthesized theranostic nanoparticles (PTCG NPs) for synergistic cascade cancer chemotherapy and chemo-dynamic therapy using polyphenol-modified block copolymer, phenolic platinum (IV) prodrug, and epigallocatechin-3-gallate (**Figure 19A**) 82. The cleavage of imine linkers could effectively release phenolic platinum (IV) prodrug in acid tumor microenvironment in HepG2 cells. Cisplatin generated by phenolic platinum (IV) prodrug elevated the H_2_O_2_ level through cascade reactions inside cells. Then Fe^III^ catalyzed to produce toxic ROS, thus boosting the therapeutic efficacy by introducing CDT. Similarly, to overcome the low Fenton reaction efficiency and insufficient H_2_O_2_ levels in TME, Fe^III^, TA, glucose oxidase (GOx), human serum albumin (HSA) and tirapazamine (TPZ) were used to synthesize a self-amplified nanoreactor termed HSA-GOx-TPZ-Fe^III^-TA (HGTFT). The nanoreactor HGTFT could realize starvation therapy by consuming oxygen, CDT via converting oxygen into ·OH for CDT, and TPZ radical-mediated chemotherapy with one injection, indicating a sustainable cascade antitumor performance 83.

Recently, inspired by the ROS-producing and ferroptosis-inducing agents for CDT, Wu et al. prepared MPN nanocomplexes based on Fe^III^ and TA self-assembly coordination chemistry to counter drug resistance through a ferroptosis/apoptosis hybrid approach (**Figure 19B**) 16. Using deferoxamine and ferrostatin-1 for the cytotoxicity assay, it was shown that the DOX-induced apoptosis was necessary for the anti-tumor activity of DTF nanocomplexes, indicating that the DDTF-mediated ferroptosis was instigated by DOX-induced apoptosis. The *in vivo* results showed that the increased ROS concentration generated by DOX-induced apoptosis could make cancer cells more sensitive to Fenton reaction-induced ferroptosis, improving the chemo-dynamic therapy effect.

Similar to CDT, photo-dynamic therapy (PDT) is another efficient strategy to eliminate tumor cells via ROS-like singlet oxygen (*^1^O_2_*) induced by photosensitizers (**Figure 19C**) 84. Therefore, much effort has been invested in photosensitizer design, targeted delivery, and controlled release for PDT., For example, Caruso's group reported a strategy to deliver photosensitizers to cancer cells by utilizing pH-sensitive polyethylene glycol metal-phenolic network (PEG-MPN) capsules carrying hematoporphyrin monomethyl ether 8. Using folic acid, HMME-doped PEG-MPN capsules (MPN@HMMEs) accumulated in carcinoma cells selectively, releasing HMME in the lysosomes due to the physiologically acidic tumor environment.

Besides the single treatment strategy alone, MPNs are also excellent candidates for designing multi-functional nanomaterials as combined therapeutic platforms due to their functional metal ions, various phenolic ligands, easy modification, and high efficiency for loading theranostic agents. A metal-phenolic network-based multi-functional platform (PID@Fe-TA), consisting of Fe^III^, TA, DOX and indocyanine green (ICG), was fabricated for combined treatment of breast cancer with photo-thermal therapy, chemo-dynamic therapy, and chemo-therapy (**Figure 19D**) 85. The TA in PID@Fe-TA reduced Fe^III^ to Fe^II^, which could react with intracellular H_2_O_2_ to generate ·*OH* for chemo-dynamic therapy. The photothermal conversion property of ICG under NIR 808-nm laser enabled PID@Fe-TA to generate heat and promote more ROS concentration, leading to photo-thermal therapy and enhanced chemo-dynamic therapy. Finally, with additional chemotherapy of DOX, PID@Fe-TA offered attractive pH-responsive multi-functional nanotheranostics.

## Conclusion and perspective

MPNs with simple self-assembly synthesis, excellent *in vitro* and *in vivo* biocompatibility, multi-functionality, and unique physicochemical properties are potential candidate for cancer theranostics. MPNs also act as a platform for tumor imaging, drug delivery carriers, and agents for photo-thermal therapy, radiotherapy, and chemo-/photo-dynamic therapy. The traditional theranostic nano-platforms represent sophisticated nanostructures requiring a combination of physical and/or chemical procedures for diagnostic and therapeutic components 56, resulting in increased biological toxicity. Thus, the elaborate and complex synthesis represents a major limitation for practical applications. However, the development of various simple MPNs exemplifies an innovative possibility of green and efficient theranostic nanomedicine. MPNs and the components used for their synthesis, phenolic ligands and metal ions, are routinely used in medical applications. Also, the fabrication processes including one-step and multi-step assembly, template-mediated preparation, and biphasic interfacial supramolecular self-assembly are simple, fast, and inexpensive.

An important prerequisite to realize the promising potential of theranostic metal-phenolic structures as nano-platforms is to extend their circulation time in the blood for controlled and targeted treatment 86. For new nanotechnologies, there are serious concerns about the *in vivo* metabolism and toxicity 87. Therefore, it is imperative to explore the metabolism and *in vivo* biological interactions of MPNs for their theranostic applications. MPNs have heralded new avenues for designing functional materials for cancer theranostic applications and future research is expected to further advance this field.

## Figures and Tables

**Figure 4:**
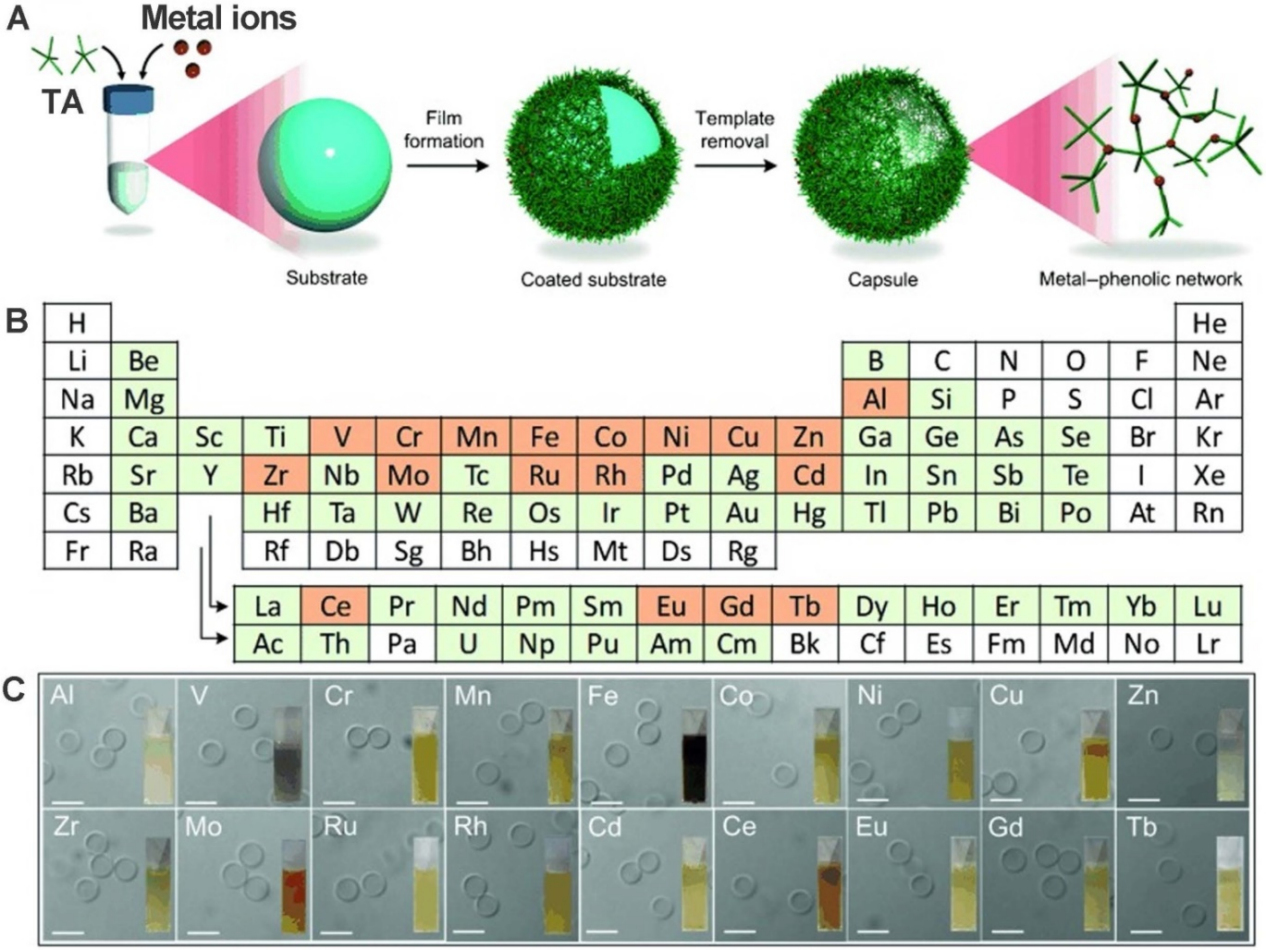
Preparation of various MPNs from different metals. a) Schematic illustrations of synthesized MPNs. b) Periodic table. c) DIC images of different MPN capsules. Scale bars are 5 µm. Adapted with permission from 25, copyright 2014 Angewandte Chemie International Edit.

**Table 1:** Various MPN systems for cancer theranostics

Metal ions	Phenolic ligands	Structure	Applications	Ref.
Fe^3+^	TA	nanoparticles	MRI, drug delivery	11
Fe^3+^	TA	hollow, hollow capsule	MRI, PTT, CDT	12,16
Fe^3+^	TA	hydrogel	PTT	14
Mg^2+^	TA	3D phenolic structure	drug delivery	46
Mn^2+^	TA	hollow, hollow capsule	MRI	12,25
Al^3+^	TA	hollow capsule	drug delivery	25,33
Ti^4+^	TA	hydrogel	drug delivery	50
Co^2+^	TA	film, hollow capsule	Catalysis	13,25
V^3+^	TA	hollow, hollow capsule	PTT	12,25
Cu^2+^	TA	hollow, hollow capsule	PET	25
Zn^2+^	TA	hollow capsule		25
Ni^2+^	TA	hollow, film	Catalysis	12,13,25
Cr^4+^	TA	hollow capsule		25
Zr^4+^	TA	hollow capsule, hydrogel	drug delivery	25,50
Mo^4+^	TA	hollow capsule		25
Rh^3+^	TA	hollow capsule	Catalysis	25
Ce2+	TA	hollow capsule		25
Eu^3+^	TA	hollow capsule	Fluorescence imaging	25
Tb^3+^	TA	hollow capsule	Fluorescence imaging	25
Cd^+^	TA	hollow capsule		25
Gd^3+^	TA	hollow, hollow capsule	MRI	12,25
Ru^3+^	TA	hollow, hollow capsule	PTT	12,25
Fe^3+^	GA	capsule, film	drug delivery	17
Fe^2+^	GA	capsule	CDT	32
Fe^3+^	PG	capsule, film	drug delivery	17
Fe^3+^	PC	capsule, film	drug delivery	17
Fe^3+^	CC	capsule	drug delivery	18
Fe^3+^	CG	capsule	drug delivery	18
Fe^3+^	Myricetin	hollow capsule		19
Fe^3+^	Quercetin	hollow capsule		19
Fe^3+^	Luteolin	hollow capsule		19
Fe^3+^	Fisetin	hollow capsule		19
Pt^2+^	EGCG	nanoparticles	Chemotherapy, CDT	82

## Abbreviations

AA: acetylacetone
CDT: chemodynamic therapy
CC: cyclodextrin catechol
CG: cyclodextrin galloyl
DPPF NPs: DOX@Pt prodrug Fe^III^ nanoparticles
DDTF: Den-DOX-tannic acid-Fe^III^ nanoparticles
Fe-CPNDs: pH-activated nanodots
GA: gallic acid
GOx: glucose oxidase
Gd: gadolinium
Hap: phenolic-functionalized hyaluronic acid
HSA: human serum albumin
MRI: magnetic resonance imaging
MPNs: metal-phenolic networks
MPN@HMMEs: hematoporphyrin monomethyl ether-doped PEG-MPN capsules
PG: pyrogallol
PC: pyrocatechol
PEGp: poly (ethylene glycol)
PSS: poly (styrene sulfonate)
PAI: photoacoustic imaging
PDT: photodynamic therapy
PD-1: programmed cell death protein 1
PTT: Photothermal therapy
RDT: Radiodynamic therapy
RT: radiotherapy
TA: tannic acid
TAA: 2-thenoyltrifluoroacetone
TME: tumor microenvironment
TPZ: tirapazamine
